# Genomic analysis of European bovine *Staphylococcus aureus* from clinical versus subclinical mastitis

**DOI:** 10.1038/s41598-020-75179-2

**Published:** 2020-10-23

**Authors:** Jurriaan Hoekstra, Aldert L. Zomer, Victor P. M. G. Rutten, Lindert Benedictus, Arjan Stegeman, Mirlin P. Spaninks, Torben W. Bennedsgaard, Andrew Biggs, Sarne De Vliegher, Demetrio Herrera Mateo, Reglindis Huber-Schlenstedt, Jørgen Katholm, Péter Kovács, Volker Krömker, Guillaume Lequeux, Paolo Moroni, Luís Pinho, Sebastian Smulski, Karlien Supré, Jantijn M. Swinkels, Mark A. Holmes, Theo J. G. M. Lam, Gerrit Koop

**Affiliations:** 1 Department Population Health Sciences, Faculty of Veterinary Medicine, University of Utrecht, Utrecht, The Netherlands; 2 Department of Basic Sciences, Faculty of Veterinary Medicine, Utrecht University, Utrecht, The Netherlands; 3 Department of Animal Science, Aarhus University, Aarhus, Denmark; 4 The Vale Veterinary Group, Tiverton, UK; 5 Department of Obstetrics, Reproduction and Herd Health, Faculty of Veterinary Medicine, University of Ghent, Ghent, Belgium; 6 Q-Llet SLP, Seva, Barcelona, Spain; 7 Bavarian Animal Health Service, Poing, Germany; 8 DNA Diagnostic, Risskov, Denmark; 9 University of Veterinary Medicine, Budapest, Hungary; 10 Department of Veterinary and Animal Sciences, University of Copenhagen, Copenhagen, Denmark; 11 LABOCEA, Fougères, France; 12 Department of Veterinary Medicine, University of Milan, Milan, Italy; 13 Department of Veterinary Clinics, Abel Salazar Biomedical Sciences Institute, University of Porto, Porto, Portugal; 14 Department of Internal Diseases and Diagnosis, Faculty of Veterinary Medicine and Animal Science, Poznan University of Life Science, Poznan, Poland; 15 MCC-Vlaanderen, Lier, Belgium; 16 MSD Animal Health, Boxmeer, The Netherlands; 17 Department of Veterinary Medicine, University of Cambridge, Cambridge, UK; 18 Royal GD, Deventer, The Netherlands

**Keywords:** Genetics, Microbiology, Diseases

## Abstract

Intramammary infections (IMI) with *Staphylococcus aureus* are a common cause of bovine mastitis and can result in both clinical (CM) or subclinical mastitis (SCM). Although bacterial isolates of *S. aureus* differ in their virulence potential it is largely unclear which bacterial virulence factors are responsible for increased clinical severity. We performed a genome wide association study and used a generalized linear mixed model to investigate the correlation between gene carriage, lineage and clinical outcome of IMI in a collection of *S. aureus* isolates from cattle with CM (n = 125) and SCM (n = 151) from 11 European countries. An additional aim was to describe the genetic variation of bovine *S. aureus* in Europa. The dominant lineages in our collection were clonal complex (CC) 151 (81/276, 29.3%), CC97 (54/276, 19.6%), CC479 (32/276, 11.6%) and CC398 (19/276, 6.9%). Virulence and antimicrobial resistance (AMR) gene carriage was highly associated with CC. Among a selection of nine virulence and AMR genes, CC151, CC479 and CC133 carried more virulence genes than other CCs, and CC398 was associated with AMR gene carriage. Whereas CC151, CC97 were widespread in Europe, CC479, CC398 and CC8 were only found in specific countries. Compared to CC151, CC479 was associated with CM rather than SCM (OR 3.62; 95% CI 1.38–9.50) and the other CCs were not. Multiple genes were associated with CM, but due to the clustering within CC of carriage of these genes, it was not possible to differentiate between the effect of gene carriage and CC on clinical outcome of IMI. Nevertheless, this study demonstrates that characterization of *S. aureus* CC and virulence genes helps to predict the likelihood of the occurrence of CM following *S. aureus* IMI and highlights the potential benefit of diagnostics tools to identify *S. aureus* CC during bovine mastitis.

## Introduction

Mastitis is responsible for significant financial losses on dairy farms due to reduced milk yield, milk unsuitable for consumption, treatment costs and culling of animals^1,2^. The main causes of bovine mastitis are bacterial intramammary infections (IMI), with *Staphylococcus aureus* being one of the most relevant pathogens^3^. Infections with *S. aureus* mostly result in subclinical mastitis (SCM), but can also lead to clinical mastitis (CM)^4^.

The *S. aureus* clones responsible for bovine mastitis predominantly belong to bovine-associated clonal complexes (CC), such as CC151, CC97, CC133, CC479 and CC771^5,6^. The genomic content of *S. aureus* clones can differ greatly due to their accessory genome, which makes up approximately 25% of the total genome^7^. The accessory genome of *S. aureus* predominantly consists of genes introduced by horizontal gene transfer (HGT), with these transferred genes often being phage genes, virulence and antimicrobial resistance (AMR) genes, expected to affect the pathogenesis of *S. aureus* IMI^8^. A large number of virulence genes have been identified in *S. aureus* isolates obtained from bovine mastitis cases, several of which are involved in evasion of the host immune response during infection^8,9^. Leukocidins, such as LukMF’, can directly attack immune cells in the lumen of the mammary gland and superantigens (SAs), such as enterotoxins *seA*–*seQ*, or toxic shock syndrome toxin 1 *(tsst-1*), massively activate T lymphocytes and antigen-presenting cells, interfering with the buildup of a proper adaptive immune response^10^. Furthermore, Staphylococcal superantigen-like proteins (SSLs) disrupt different pathways of the immune response, such as pathogen recognition by TLR-2 (SSL-3, SSL-4), neutrophil function and recruitment (SSL-1, SSL-5, SLL-7, SSL-13) and the opsonization of bacteria (SSL-7)^10,11^. Some of these immune evasion factors are considered ruminant adapted^10^, such as LukMF’ and the *S. aureus* pathogenicity island encoded variant of the Von Willebrand factor‐binding protein (SaPI *vWFbp*)^12,13^. Furthermore, *S. aureus* can carry AMR encoding genes, including *blaZ*, *tetM* and *mecA,* which reduce effectiveness of antimicrobial treatment of *S. aureus* mastitis^14^. The majority of virulence and AMR genes are located on mobile genetic elements and *S. aureus* belonging to the same CC have specific virulence gene signatures due to clonal expansion^6,15,16^ and restriction-modification systems that reduce HGT between different *S. aureus* lineages^17,18^.

Several studies investigated CCs and virulence/AMR gene carriage among bovine *S. aureus* isolates responsible for IMI^6,9,19^, but only few directly compare isolates from CM and SCM cases. Recently, we identified a specific bovine *S. aureus* lineage (CC479) that was associated with CM in The Netherlands^20^. In addition, carriage of certain genes (*lukM-lukF*′, *seO*) was overrepresented among CM isolates compared to SCM isolates^21,22^. This suggests that some *S. aureus* lineages are more likely to cause CM than others. Although both CM and SCM lead to financial losses for the farm and are associated with animal disease and welfare, the costs of treatment of CM are substantially higher than of SCM^1,23^. Also, the effect of CM on animal welfare obviously is considerable larger than for SCM^24^. Therefore, identification of *S. aureus* isolates with increased risk of developing into CM is likely valuable to better support farm health management decisions. However, studies comparing isolates from CM and SCM have been mainly on a national scale^20–22^, whereas the genetic makeup of bovine *S. aureus* likely differs between countries. Therefore, in this study we performed whole-genome sequencing (WGS) of 276 *S. aureus* isolates from cattle with CM or SCM in 11 European countries.

The primary aim of this study was to investigate differences in lineage and genomic content between *S. aureus* isolates responsible for CM and SCM. A second aim was to describe the variation in lineages, virulence gene and AMR gene carriage of bovine *S. aureus* isolates in Europe.

## Results

### Bovine *S. aureus* CCs differ in their carriage of immune evasion and AMR genes

After selection of isolates and quality control of WGS results, 276 genomes were available of *S. aureus* isolates obtained from bovine mastitis cases originating from 254 unique herds in eleven different European countries. There was an approximately even distribution of CM (125/276, 45%) and SCM (151/276, 55%) isolates, and *S. aureus* in the collection belonged to eighteen different CCs. The most prevalent CCs were CC151 (81/276, 29.3%), CC97 (54/276, 19.6%), CC479 (32/276, 11.6%), CC133 (25/276, 9.1%), CC398 (19/276, 6.9%), CC1 (14/276, 5.1%), CC20 (11/276, 4.0%) and CC8 (11/276, 4.0%), and carriage of a selection of nine key virulence and AMR genes among isolates was related to CC (Table 1). The CC151, CC479 and CC133 strains had high carriage of virulence genes but lacked AMR genes. All these three CCs carried the *lukM-lukF′* genes and CC479, CC151 also possessed SA genes. In addition, CC479, CC133 *S. aureus* carried the SaPI encoded *vWFbp* gene (Table 1). In contrast, CC398 *S. aureus* lacked all these virulence factors but did have a high carriage rate of the AMR genes *blaZ* (8/19, 42%), *tetM* (19/19, 100%) and *mecA* (11/19, 58%) (Table 1). The CC97 displayed a moderate carriage of both virulence gene *lukM-lukF’* (16/54, 30%) and the AMR gene *blaZ* (16/54, 30%) (Table 1).

**Table 1:** Number and percentage of isolates positive for a selection of virulence and antimicrobial resistance genes per clonal complex and number and proportion of clinical mastitis of 276 *S. aureus* isolates obtained from bovine clinical and subclinical mastitis in 11 European countries.

CC^a^	n	*%*	Virulence genes n (%)						Antimicrobial resistance genes n (%)			Manifestation of mastitis		
			*lukM-lukF′*^b^	*scn*	SaPI *vWFbp*	*seI*	*seL*	*tsst-1*	*blaZ*	*tetM*	*mecA*	CM^c^	Odds ratio^d^ (95% CI)	P^g^
151	81	29.3	81 (100)	0 (0)	0 (0)	80 (99)	19 (23)	19 (23)	1 (1)	0 (0)	0 (0)	41 (51)	Ref^e^	Ref
97	54	19.6	16 (30)	7 (13)	0 (0)	0 (0)	0 (0)	0 (0)	16 (30)	0 (0)	0 (0)	23 (42)	0.75 (0.37–1.54)	0.44
479	32	11.6	30 (94)	0 (0)	32 (100)	32 (100)	0 (0)	0 (0)	0 (0)	1 (3)	0 (0)	25 (78)	3.62 (1.38–9.50)	< 0.01
133	25	9.1	23 (92)	21 (84)	25 (100)	0 (0)	2 (8)	2 (8)	0 (0)	0 (0)	0 (0)	13 (52)	1.05 (0.42–2.61)	0.92
398	19	6.9	0 (0)	10 (52)	0 (0)	0 (0)	0 (0)	0 (0)	8 (42)	19 (100)	11 (58)	5 (27)	0.41 (0.12–1.48)	0.17
1	14	5.1	9 (64)	0 (0)	0 (0)	0 (0)	0 (0)	0 (0)	0 (0)	0 (0)	0 (0)	3 (21)	0.30 (0.07–1.23	0.10
20	11	4.0	0 (0)	0 (0)	0 (0)	7 (64)	0 (0)	0 (0)	0 (0)	0 (0)	0 (0)	0 (0)	0.34 (0.08–1.45)	0.15
8	11	4.0	0 (0)	0 (0)	0 (0)	0 (0)	0 (0)	0 (0)	6 (54)	0 (0)	0 (0)	2 (18)	0.21 (0.43–1.10)	0.06
9	5	1.8	0 (0)	0 (0)	0 (0)	0 (0)	0 (0)	0 (0)	4 (66)	0 (0)	0 (0)	2 (33)	NA^f^	NA
50	5	1.8	0 (0)	0 (0)	0 (0)	0 (0)	0 (0)	0 (0)	1 (20)	0 (0)	0 (0)	1 (20)	NA	NA
49	4	1.4	1 (25)	0 (0)	0 (0)	0 (0)	1 (25)	0 (0)	1 (25)	0 (0)	0 (0)	1 (25)	NA	NA
7	4	1.4	0 (0)	0 (0)	0 (0)	0 (0)	0 (0)	0 (0)	0 (0)	0 (0)	0 (0)	2 (50)	NA	NA
5	4	1.4	0 (0)	0 (0)	0 (0)	4 (100)	0 (0)	0 (0)	0 (0)	0 (0)	0 (0)	3 (75)	NA	NA
45	3	1.1	0 (0)	0 (0)	0 (0)	1 (33)	1 (25)	0 (0)	1 (33)	0 (0)	0 (0)	0 (0)	NA	NA
101	1	0.4	0 (0)	0 (0)	0 (0)	0 (0)	0 (0)	0 (0)	0 (0)	0 (0)	0 (0)	0 (0)	NA	NA
22	1	0.4	0 (0)	0 (0)	0 (0)	1 (100)	0 (0)	0 (0)	1 (100)	0 (0)	0 (0)	0 (0)	NA	NA
30	1	0.4	0 (0)	0 (0)	0 (0)	0 (0)	0 (0)	0 (0)	1 (100)	0 (0)	0 (0)	0 (0)	NA	NA
425	1	0.4	0 (0)	0 (0)	0 (0)	0 (0)	0 (0)	0 (0)	0 (0)	0 (0)	0 (0)	1 (100)	NA	NA
Total	276		160 (58)	38 (14)	57 (21)	125 (45)	24 (9)	21 (8)	40 (15)	20 (7)	11 (4)	125 (45)		

^a^Clonal complex.

^b^Carriage of genes determined using pangenome data from *roary.*

^c^Number and percentage of clinical mastitis cases per CC.

^d^Cluster-specific odds ratio of the isolate being cultured from CM versus SCM from a generalized linear mixed model with country as a random effect.

^e^Reference class for variable CC within the generalized linear mixed model.

^f^Only CCs with n > 10 were included in model.

^g^Significance of class within the variable CC.

Heatmaps of the BLAST score ratio (BSR)^25^ of all *S. aureus* genes annotated as (putative) SAs or SSLs by *prokka*^26^ demonstrated that bovine *S. aureus* CCs differ in their carriage of these immune evasion factors (Supplementary Figs. S1, S2). Notable differences in SA carriage were the high number of SAs (up to 12 different SAs) genes carried by CC151, whereas CC398 isolates lacked SAs. Although most SSLs were detected in all *S. aureus*, the BSR of these SSLs differed between *S. aureus* CCs. However, the SSL-7, SSL-8, SSL-9 genes were only absent in CC479 *S. aureus*. Furthermore, two genes that were annotated as unnamed SSLs (GenBank references: WP_143564871.1 and WP_124375191) were exclusively found among CC97 isolates (Supplementary Fig. S2).

To screen for target genes that could differentiate between major ruminant CCs in a PCR-based assay, the presence of potential CC exclusive genes was also investigated (Supplementary Fig. S3, Supplementary Table S1). The highest number of CC-exclusive genes were found for CC479 (n = 17), followed by CC20 (n = 4), CC151 (n = 3), CC8 (n = 2) and CC133 (n = 2). For CC97, CC398, only a single unique gene was identified and no CC exclusive genes were found for CC1 isolates.

### Heterogeneous spatial distribution of CCs across Europe

There was a significant difference in the distribution of *S. aureus* CCs between different countries (Fisher's Exact Test, p < 0.001). Whereas CC151 (10 out 11 countries) and CC97 (9 out 11) *S. aureus* were detected in almost all countries, CC398 (6 out 11) and CC479 (5 out 11) were considerably less widespread in our collection (Table 2). The CC398 lineage was predominantly found in isolates from Poland and Spain. Isolates belonging to CC8 originated exclusively from either Italy or Bavaria region in Germany. Also, all CC50 isolates originated from Denmark (Table 2).

**Table 2:** Number and percentage of bovine *S. aureus* of 276 *S. aureus* isolates per clonal complex ranked per country or contributing region obtained from bovine clinical and subclinical mastitis in 11 European countries.

CC^a^	N (%)	Be n (%)	Dk n (%)	Fr n (%)	Ge (Ls^b^) n (%)	Ge (By^c^) n (%)	Hu n (%)	It n (%)	NL n (%)	Po n (%)	Pt n (%)	Es n (%)	Uk n (%)
151	81 (29)	13 (43)	5 (31)	4 (17)	10 (34)	8 (27)	3 (16)	6 (20)	14 (64)	0 (0)	2 (29)	2 (17)	14 (52)
97	54 (20)	4 (13)	1 (6)	7 (30)	4 (14)	6 (20)	7 (37)	9 (30)	0 (0)	5 (16)	0 (0)	2 (17)	9 (33)
479	32 (12)	8 (27)	0 (0)	0 (0)	2 (7)	8 (27)	0 (0)	3 (10)	8 (36)	0 (0)	3 (43)	0 (0)	0 (0)
133	25 (9)	4 (13)	4 (25)	2 (9)	7 (24)	2 (7)	0 (0)	2 (7)	0 (0)	1 (3)	0 (0)	3 (25)	0 (0)
398	19 (7)	0 (0)	0 (0)	1 (4)	2 (7)	0 (0)	1 (5)	1 (3)	0 (0)	10 (32)	0 (0)	4 (33)	0 (0)
1	14 (5)	1 (3)	0 (0)	1 (4)	0 (0)	0 (0)	2 (11)	1 (3)	0 (0)	7 (23)	0 (0)	0 (0)	2 (7)
20	11 (4)	0 (0)	0 (0)	4 (17)	1 (3)	0 (0)	5 (26)	0 (0)	0 (0)	0 (0)	0 (0)	1 (8)	0 (0)
8	11 (4)	0 (0)	0 (0)	0 (0)	0 (0)	5 (17)	0 (0)	6 (20)	0 (0)	0 (0)	0 (0)	0 (0)	0 (0)
9	5 (2)	0 (0)	1 (6)	0 (0)	0 (0)	0 (0)	0 (0)	1 (3)	0 (0)	1 (3)	2 (29)	0 (0)	0 (0)
50	5 (2)	0 (0)	5 (31)	0 (0)	0 (0)	0 (0)	0 (0)	0 (0)	0 (0)	0 (0)	0 (0)	0 (0)	0 (0)
5	4 (1)	0 (0)	0 (0)	2 (9)	0 (0)	0 (0)	0 (0)	1 (3)	0 (0)	0 (0)	0 (0)	0 (0)	1 (4)
49	4 (1)	0 (0)	0 (0)	1 (4)	0 (0)	0 (0)	0 (0)	0 (0)	0 (0)	3 (10)	0 (0)	0 (0)	0 (0)
7	4 (1)	0 (0)	0 (0)	0 (0)	3 (10)	0 (0)	1 (5)	0 (0)	0 (0)	0 (0)	0 (0)	0 (0)	0 (0)
45	3 (1)	0 (0)	0 (0)	1 (4)	0 (0)	0 (0)	0 (0)	0 (0)	0 (0)	2 (6)	0 (0)	0 (0)	0 (0)
101	1 (0.5)	0 (0)	0 (0)	0 (0)	0 (0)	1 (3)	0 (0)	0 (0)	0 (0)	0 (0)	0 (0)	0 (0)	0 (0)
22	1 (0.5)	0 (0)	0 (0)	0 (0)	0 (0)	0 (0)	0 (0)	0 (0)	0 (0)	1 (3)	0 (0)	0 (0)	0 (0)
30	1 (0.5)	0 (0)	0 (0)	0 (0)	0 (0)	0 (0)	0 (0)	0 (0)	0 (0)	1 (3)	0 (0)	0 (0)	0 (0)
425	1 (0.5)	0 (0)	0 (0)	0 (0)	0 (0)	0 (0)	0 (0)	0 (0)	0 (0)	0 (0)	0 (0)	0 (0)	1 (4)
Total	276	30	16	24	29	30	19	30	22	31	7	12	27

^a^Clonal complex.

^b^Lower Saxony region in Germany.

^c^Bavaria region in Germany.

### Pangenome of bovine *S. aureus* and phylogenetic analysis

**Figure 1:**
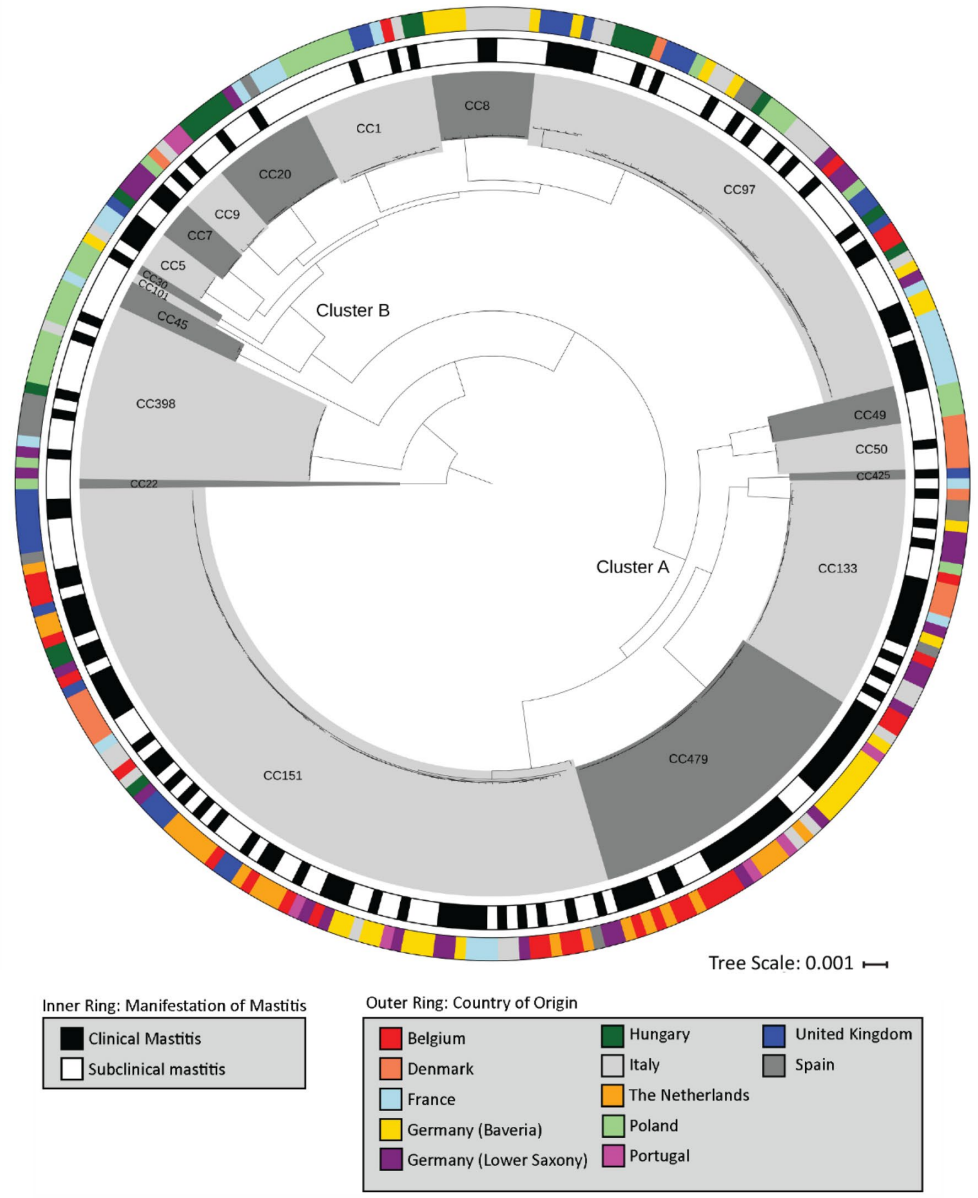
Maximum-likelihood phylogenetic tree based on the alignment of core genome (genes n = 1953) performed using *roary* v3.12^39^ and MAFFT of 276 *S. aureus* isolates obtained from bovine clinical and subclinical mastitis cases from 11 European countries. Dark and light grey shading displays Clonal Complex (CC), the outer ring represents the country of origin and the inner ring the clinical versus subclinical mastitis of each isolate. The phylogenetic tree was rooted with the CC22 clade and visualized using iTOL v3.6^41^.

**Figure 2:**
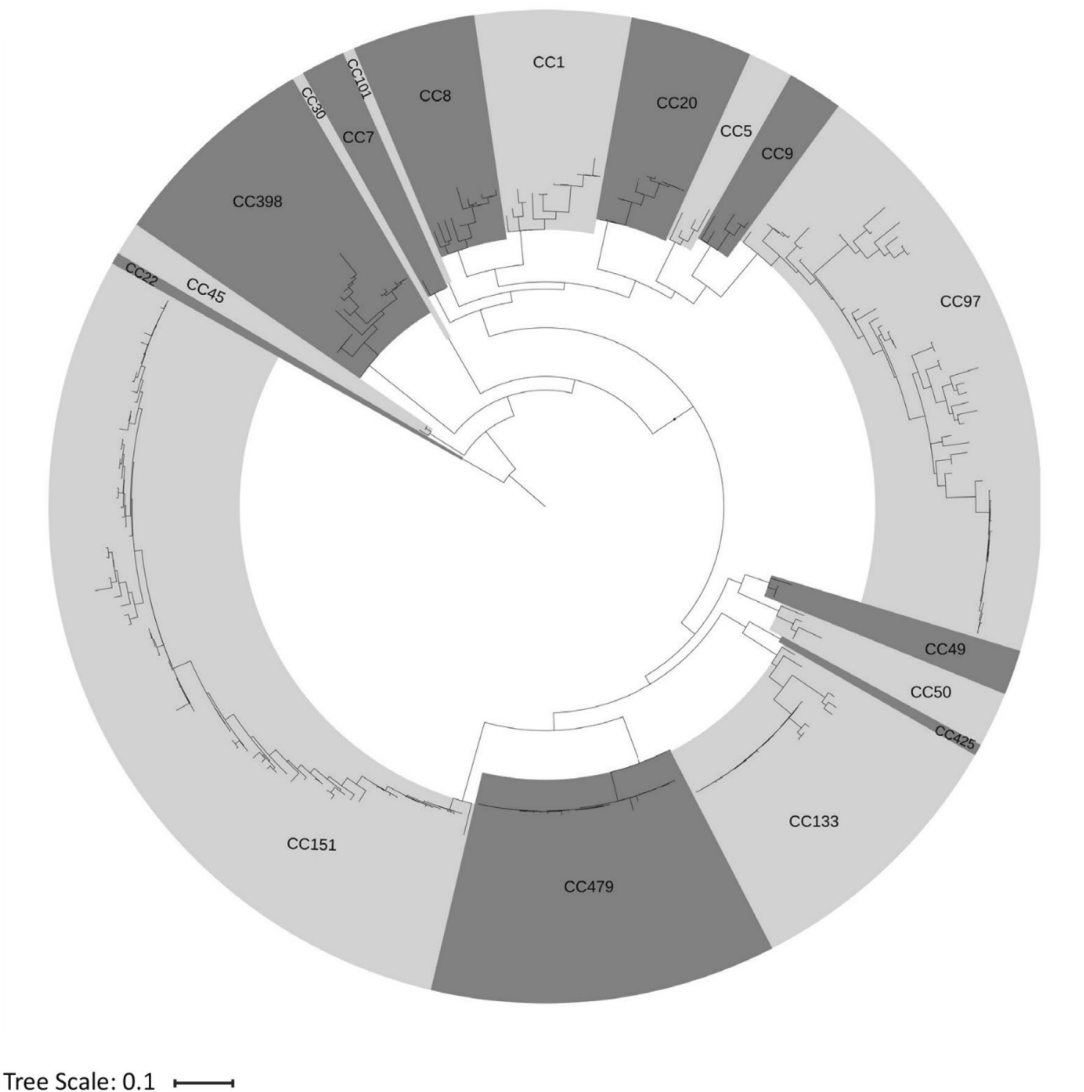
Maximum-likelihood phylogenetic tree based on presence or absence of genes of the accessory genome (n = 2023 genes) of 276 *S. aureus* isolates obtained from bovine clinical or subclinical mastitis from 11 European countries. Dark and light grey shading displays Clonal Complex (CC). The phylogenetic tree was rooted with the CC22 clade and visualized using iTOL v3.6^41^.

To investigate the phylogeny of our bovine *S. aureus* isolates, the pangenome of the entire collection was determined (n = 5720 genes) and a phylogenetic tree was constructed based on a super-alignment of the 1953 genes in the core genome (genes present in > 99% of isolates) (Fig. 1). A second phylogenetic tree was built based on the binary presence or absence of genes in the accessory genome (Fig. 2). Within both phylogenetic trees, isolates clustered within CC and two major clusters of CCs could be identified. The largest cluster, labeled as cluster A consisted of CC151, CC479, CC133, CC425, CC49 and CC50 isolates and second largest cluster B included CC97, CC8, CC1, CC20, CC9, CC7, CC5, CC30 and CC101. (Figs. 1, 2). There was uneven distribution of CM and SCM isolates between clusters, with more CM isolates being present in Cluster A (81/148, 55%) compared to cluster B (38/103, 36%) (Pearson's Chi-squared test p < 0.01).

In general, the phylogenetic tree based on the core genome was similar to the tree based on the accessory genome. In addition, the phylogenetic tree shows considerable variation in accessory gene content, most notably within CC151 and CC97, but almost no variation in accessory gene carriage was observed among CC479 and CC133 *S. aureus* isolates (Fig. 2).

### *Staphylococcus aureus* CC479 is associated with CM

In order to further study the association between *S. aureus* CC and clinical outcome of IMI, a generalised linear mixed model (GLMM) was built with CM versus SCM as the outcome variable, CC as predictor variable and the country of orgin of isolates as a random effect. For this model, isolates belonging to CCs with n < 10 (CC9, CC50, CC49, CC7, CC45, CC5, CC101, CC22, CC30, and CC425) were clustered together in a single category labeled as ‘other’. Including CC improved the fit over the model compared to the model with only random effects (ANOVA, p = 0.003) and isolates belonging to CC479 were more likely to originate from CM than from SCM (OR 3.62; 95% CI 1.38–9.50, p < 0.01) compared to the reference category (CC151). In addition, there was a trend for CC8 (OR 0.22; 95% CI 0.04–1.1; p = 0.06) and CC1 (OR 0.30; 95% CI 0.07–1.23; p = 0.10) to originate from SCM (Table 1).

### Clinical manifestation of mastitis is associated with a large number of different genes

To study genetic differences that could underlay variation in pathogenity of bovine *S. aureus* isolates, we performed a genome wide association study (GWAS) and 153 genes were associated with CM isolates. In agreement with the results from the GLMM, genes exclusively present in the CC479 isolates (n = 59) had the highest OR, ranging from 4.6 to 5.6 [Benjamini–Hochberg Procedure (BHP); p-values < 0.05]. Among the CM-associated genes, a total of 20 genes matched our inclusion criteria for significant genes of interest (best pairwise comparision p < 0.1; BHP p < 0.1) and the OR of these genes ranged from 1.95 to 3.42 (Table 3). Among these genes, the hypothetical potentially bacteriophage derived DUF3310 domain-containing protein (OR 2.35) was the only gene associated with CM that was detected among all main lineages. Carriage of SA genes *seM*, *seN*, *seI*, *seG*, *seO* and *seU*, as well as the accessory gene regulator (*agr*) D type II gene was associated with CM and these genes were present in all CC479 isolates and the majority of CC151 isolates (Table 3). In addition, three genes from within the *S. aureus* pathogenicity island bovine 3 (SaPI_bov3_) were also associated with CM and were carried by all CC151, CC479 and 77% of CC97 isolates.

**Table 3:** Odds Ratio, carriage rate per clonal complex and GenBank references of genes associated with clinical mastitis based on a genome wide association study performed on 276 *S. aureus* isolates obtained from bovine clinical and subclinical mastitis in 11 European countries.

Gene^a^	Odds Ratio^b^	Best pairwise comparision p^c^	CC151 (%)^d^	CC97 (%)	CC479 (%)	CC133 (%)	CC398 (%)	CC1 (%)	CC20 (%)	CC8 (%)	Other CCs (%)^5^	Weighted average (%)	GenBank reference
DUF3310 domain-containing protein	2.35	< 0.001	82	60	97	74	95	79	80	30	50	75	WP_001624706.1
DUF2483 domain-containing phage protein	2.36	0.01	43	35	97	12	0	7	0	11	10	33	WP_001077279.1
NAD-specific glutamate dehydrogenase	2.83	0.01	100	0	97	60	0	0	0	0	30	50	SAO39934.1
phiPVL ORF050-like protein	2.23	0.02	98	77	100	0	0	14	30	0	17	59	ABD21348.1
Staphylococcal enterotoxin type C1/U	2.21	0.03	98	0	100	0	0	0	0	0	66	44	WP_109162118.1
Staphylococcal enterotoxin type G	1.95	0.04	98	0	100	0	0	0	100	0	43	49	WP_141060424.1
Staphylococcal enterotoxin type N	1.95	0.04	98	0	100	0	0	0	100	0	43	49	WP_109162119.1
Staphylococcal enterotoxin type I	1.95	0.04	98	0	100	0	0	0	100	0	43	49	QCW39073.1
Multidrug transporter protein (SaPI_bov3_)	2.31	0.04	99	77	100	0	0	14	30	0	17	59	WP_065315972.1
Intramembrane metalloprotease/(SaPI_bov3_)	2.31	0.04	99	77	100	0	0	14	30	0	17	59	WP_070008570.1
Hypothetical protein (SaPI_bov3_)	2.31	0.04	99	77	100	0	0	14	30	0	17	59	WP_000921697.1
Staphylococcal enterotoxin type M	2.07	0.06	98	0	100	0	0	0	0	0	20	43	WP_109162116.1
SAS066 AgrD ( type II)	2.52	0.06	98	0	100	0	0	0	0	0	47	46	SCU54394.1
Accessory gene regulator protein B4 (type II)	2.52	0.06	98	0	100	0	0	0	0	0	47	46	CZQ66246.1
Hypothetical protein	2.83	0.06	100	13	100	0	0	0	0	0	3	44	WP_000389772.1
TfoX/Sxy family protein	2.83	0.06	100	13	100	0	0	0	0	0	3	44	WP_000179903.1
Arsenate reductase	1.97	0.06	99	0	100	100	0	0	0	100	66	61	WP_000163240.1
Trypsin-like serine protease	2.61	0.06	0	0	100	0	0	0	0	0	50	17	WP_043054986.1
SACOL0901 pathogenicity island protein	2.82	0.07	98	88	100	84	0	14	30	28	27	71	WP_109183239.1
DUF1433 domain-containing protein	3.42	0.07	98	98	100	100	0	0	100	0	63	79	WP_031900638.1

^a^Gene annotation by *prokka* and confirmed using BLAST search of gene sequence 4. Carriage rate of gene per clonal complex.

^b^Odds Ratio of causing clinical rather than subclinical mastitis from the Genome wide association study 5. Includes isolates belonging to CC9, CC50, CC5, CC49, CC7, CC45, CC101, CC20, CC30 and CC425.

^c^Best pairwise comparison P value from GWAS performed using scoary.

Furthermore, 61 genes were associated with SCM (i.e. an OR < 1), from which 10 genes matched our selection criteria and the OR of these genes ranged between 0.09 and 0.44 (Table 4). Most genes (8/10) coded for hypothetical proteins and the genes with predicted function were identified as an antitoxin YezG family protein (OR 0.32) and a putative DNA binding protein (OR 0.29). The SCM-associated genes were mostly carried by CC1, CC20, and CC8 *S. aureus*, but were always absent from CC479 and most CC151 isolates (Table 4).

**Table 4:** Odds Ratio, carriage rate per clonal complex and GenBank references of genes associated with subclinical mastitis based on a genome wide association study performed on 276 *S. aureus* isolates obtained from bovine clinical and subclinical mastitis in 11 European countries**.**

Gene^a^	Odds ratio^b^	Best pairwise comparision p^c^	CC151 (%)^d^	CC97 (%)	CC479 (%)	CC133 (%)	CC398 (%)	CC1 (%)	CC20 (%)	CC8 (%)	Other CCs (%)^e^	Weighted average (%)	GenBank reference
Putative DNA-binding protein	0.29	< 0.01	2	22	0	16	42	64	82	18	7	18	AUM57693.1
Hypothetical protein	0.40	< 0.01	0	94	0	0	100	100	100	100	34	42	WP_000375476.1
Hypothetical protein	0.27	0.02	0	0	0	0	100	100	100	100	34	24	WP_000070812.1
Probable antitoxin YezG	0.32	0.03	0	0	0	100	0	0	100	0	3	15	WP_000142094.1
Hypothetical protein	0.38	0.03	0	0	0	0	100	100	100	100	62	27	WP_078068548.1
Hypothetical protein	0.44	0.03	0	98	0	0	0	100	100	100	62	39	WP_072426418.1
Hypothetical protein	0.32	0.04	0	50	0	0	100	7	0	91	52	26	WP_000431307.1
Hypothetical protein	0.09	0.06	0	0	0	8	32	0	0	9	14	5	WP_000993183.1
Hypothetical protein	0.26	0.06	0	0	0	0	0	100	0	100	34	13	ETO57257.1
Hypothetical protein	0.43	0.06	1	0	0	100	100	100	100	100	86	39	WP_078370397.1

^a^Gene annotation by *prokka* and confirmed using BLAST search of gene sequence.

^b^Odds Ratio of causing clinical rather than subclinical mastitis from the Genome wide association study.

^c^Best pairwise comparison P value from GWAS performed using scoary.

^d^Carriage rate of gene per clonal complex.

^e^Includes isolates belonging to CC9, CC50, CC5, CC49, CC7, CC45, CC101, CC20, CC30 and CC425.

## Discussion

There is a large diversity in the carriage of virulence and AMR genes between bovine *S. aureus* lineages^6,9^, but it is unclear which genetic differences underly the observed variation in pathogenicity following bovine IMI. Therefore, this study aimed to identify genetic differences between *S. aureus* isolated from CM and SCM in dairy cattle. A secondary goal of the study was to describe the diversity of bovine *S. aureus* lineages in Europe and their carriage of immune evasion factors. We found CC479 to be strongly associated with CM rather than with SCM cases.

Although eighteen different CCs were present in our isolate collection, most *S. aureus* belonged to a limited number of CCs, with the five CCs (CC151, CC97, CC479, CC133 and CC398) making up more than 75% of all isolates. All these CCs have previously been associated with bovine mastitis^6,19,27,28^. The distribution of *S. aureus* CCs differed between geographical locations. Although the isolates in our collection were not a random sample, they did originate from 254 unique herds with a maximum of one CM and one SCM isolate from the same herd. The prevalence of *S. aureus* CCs per country from our study is in line with studies performed in Denmark^29^, Germany^6^ and The Netherlands^30^. Nevertheless, these isolates cannot be expected to fully represent the genetic diversity of bovine *S. aureus* on a national level. Because in addition to a relative low number of isolates, there likely was some clustering within certain regions in several of the countries, which may have resulted in an underestimation of the true genetic diversity in the population. We must also note that the aim of our sampling design was to collect isolates from an equal number of clinical and subclinical cases. This does not reflect the true population of *S. aureus* isolates in dairy herds, as the prevalence of subclinical infections is substantially higher than of clinical infections. Since CM and SCM are both expressions of *S. aureus* IMI and as SCM can develop into CM and vice versa^31^, isolates may have been misclassified, biasing any associations between CM versus SCM and *S. aureus* genotype towards the null-effect. Still, our study, as well as other studies using a similar classification of mastitis^20,21^, did identify genetic differences between CM and SCM *S. aureus* isolates, but possibly, some other associations may have been missed in our approach.

A key finding of the current study was the association between *S. aureus* belonging to CC479 and CM. This corresponds with a previous study which reported that experimental infection with CC479 *S. aureus* results in more severe clinical signs and a higher bacterial load than infection with a CC151 *S. aureus* strain^32^. In addition, the association between CC479 and CM was also observed in our previous analysis of Dutch mastitis isolates^20^. The underlaying mechanisms for this apparent increased virulence of CC479 remains unknown, but we have suggested a SNP in the repressor of toxins (*rot*) gene, resulting in an increased production of LukMF’ by CC479 isolates as a possible cause^20^. Our GWAS identified several genes associated with clinical outcome of IMI, but as *S. aureus* belonging to the same CC share a similar (virulence) gene profile, there was a strong link between carriage of these genes and lineage. Therefore, it was not possible to differentiate between the effect of individual genes/sets of genes and *S. aureus* lineage on the clinical outcome of IMI. Interestingly, genes associated with CM by GWAS which were all present in CC479 isolates, were also almost all present in CC151 *S. aureus* (Table 3), whereas the latter CC had an approximately even distribution of isolates originating from CM (51%) and SCM (49%). Since CC151 and CC479 share most CM-associated genes, these genes are likely spuriously associated to CM, due to confounding by other factors within CC479. Differences in gene expression are more likely causes of the increased virulence of CC479, in line with our hypothesis regarding the non-functional *rot* gene^18^. We confirmed that this mutation was present in all CC479 isolates within our collection (results not shown). It is likely that the absence of functional *rot* affects the expression levels of multiple virulence genes within CC479 *S. aureus*^20^, likely leading to substantially increased probability of causing CM. Future research, e.g. infection studies with genetically modified CC479 isolates lacking potential virulence factors or with restored *ro*t function, are required to identify these potential causal mechanisms.

The results from our GLMM suggest that CC8 and CC1 are less likely to cause CM in cows and most of the genes that were associated with CM were carried by CC8 and CC1 isolates*.* Previous work identified that CC8 predominantly make up the *S. aureus* genotype B, a highly contagious subtype of *S. aureus*^28,33^. We only detected CC8 *S. aureus* among isolates from Italy and Germany, suggesting that this lineage is not widespread throughout Europe. Indeed, reports on CC8/GTB *S. aureus* primarily originate from Switzerland and Austria^28,34,35^. In contrast, the CC1 lineage was detected in six different countries in this study, although 50% of the CC1 isolates were collected in Poland.

Phylogenetic trees constructed based on core genome alignment and binary presence and absence of accessory genes clustered the isolates perfectly within the assigned CC. This demonstrates that MLST is an adequate genotyping technique, but Fig. 2 shows that considerable variation exists in the accessory genome among isolates within a CC. Interestingly, there was very limited variation in accessory gene carriage among CC479 and CC133 *S. aureus* isolates and this suggests a rapid clonal expansion of these specific *S. aureus* clones. The CC133 lineage is considered a ruminant-adapted lineage but is mostly associated with small ruminants^16^. Therefore, it is possible that the highly similar bovine CC133 *S. aureus* isolates represent a subgroup of CC133 *S. aureus* that is adapted to cattle and this subgroup, as well as the highly similar CC479 isolates, is considerably more successful at infecting the bovine mammary gland than other CC133, CC479 *S. aureus*.

Although our analyses showed associations between specific genes and CC with CM, it is important to note that none of these genes were essential for CM in our collection. Clones that were associated with CM cases based on CC or gene carriage were also cultured from SCM and vice versa. Besides pathogen factors, the clinical outcome of an IMI depends on host factors, such as breed, stage of lactation, environmental factors and their interaction^31,36^. Nevertheless, from a diagnostic perspective, the identification of *S. aureus* isolates with an increased risk of developing into CM (e.g. CC479) or highly contagious isolates (e.g. CC8) can support farmers and veterinarians in deciding on the most appropriate intervention strategy to control *S. aureus* in a herd. For *Streptococcus uberis,* it has been demonstrated that MALDI-TOF MS performed on milk samples can identify *S. uberis* isolates with increased CM risk^37^. However, it is not certain if this technique is able to distinguish between different CCs of *S. aureus*. There are several PCR based test that detect pathogen-specific genes in milk and an assay for the detection of CC8 (genotype B) *S. aureus* has already been developed^44^. We identified multiple CC-specific genes that could be employed to differentiate between common ruminant-associated *S. aureus* lineages (Supplementary Table S1).

In summary, this study identified that a limited number of *S. aureus* CCs are responsible for bovine mastitis in Europe and that CC479 is strongly associated with CM. Although our analysis shows specific genes are associated with CM, the presence of these genes in CC associated with SCM indicates it is likely that differential gene expression rather than gene carriage affects clinical presentation of IMI. This study shows that the type of infecting *S. aureus* influences the clinical outcome of IMI in dairy cattle. Therefore, identification of *S. aureus* CC can help predict the likelihood of the occurrence of CM following *S. aureus* IMI and highlights the potential benefit of diagnostics tools to identify *S. aureus* CC during bovine mastitis.

## Methods

### Bovine mastitis isolates

Twelve mastitis research groups or diagnostic labs from eleven countries (Belgium, Denmark, France, Germany, Hungary, Italy, Poland, Portugal, Spain, The Netherlands and United Kingdom) were asked to submit a convenience sample of approximately 30 *S. aureus* isolates obtained from cases of bovine mastitis. Participants were asked to submit a maximum of one CM and one SCM isolate per herd with as much as possible an equal number of CM and SCM isolates, coming from different regions in their country. The sample size was based on what was considered feasible to be collected within a reasonable period of time, while giving a good impression on the situation in a country. No formal sample size calculations were performed. For each isolate the sampling date, farm ID, geographical location of farm (city and/or region), cow ID, clinical manifestation (CM/SCM) was reported in a pre-structured Excel workbook (Microsoft Corp., Redmond, WA, USA).

Clinical mastitis was defined as visible signs of inflammation of the udder, indicated by a hard swollen quarter and/or abnormal milk; SCM was defined as a high somatic cell count (> 200,000 cells/mL) while no clinical signs of mastitis could be observed.. The definitions were communicated to all participating researchers and were stated in a workbook to ensure that a uniform definition of CM and SCM was applied by all participants. No constraints were put on duration or chronicity of infections. The clinical status (CM or SCM) was observed and recorded at the moment of sampling.

Isolates were recultured from their transport media onto to sheep blood agar plates, and incubated overnight at 37 °C. Next, single colonies were picked and grown in 2 mL Todd Hewitt Broth (THB) (Sigma, St. Louis, MO, USA) for 16 h at 37 °C with agitation. Bacterial glycerol stocks (25% glycerol) were made by adding 0.5 mL of bacterial broth to 0.5 mL 50% glycerol solution in distilled water. Furthermore, DNA was extracted using a simple boiling protocol to confirm bacterial species by PCR targeting the *S. aureus* specific *femA* gene, as described by Hoekstra et al*.*^20^*.* Isolates that were negative for the *femA* PCR or lacked mandatory metadata were excluded from the final collection. For each herd, a maximum of one isolate from a CM case and one from a SCM case was allowed and if multiple CM or SCM isolates were donated from the same herd, one was selected using the random number function of Excel 2015 (Microsoft, Redmond, WA, USA).

### DNA extraction, genome sequencing and multilocus sequence typing

DNA for whole genome sequencing was extracted using the DNeasy UltraClean Microbial Kit (Qiagen, Venlo, The Netherlands) according to the manufacturer’s instructions. Purity and DNA yield were measured by spectrophotometry and whole-genome sequencing was performed using Illumina HiSeq sequencing (Illumina Inc., San Diego, CA, United States). Multilocus sequence type was determined based on genome data. Each sequence type was assignment to a CC based on eBURST analysis using PHYLOViZ Online^38^.

### Annotation of genomes, pan-genome analysis and phylogenetic analyses

After quality control of whole genome sequence results, genomes were annotated using *prokka* v1.11^26^ and the pan/core genome (genes present in 99% < of genomes) determined using *roary* v3.12^39^*.* Alignment of the core genome was performed using *MAFFT* v7.407 and the phylogenetic trees was built with *Fasttree* v2.1^40^. Subsequently, trees were visualized using iTOL v3.6^41^ and trees were rooted using the CC22 clade. The large-scale BLAST score ratio (LS-BSR) pipeline was used to obtain matrixes with BLAST score ratio of each annotated gene^25^. For each isolate, the presence of the genes encoding leukocidin LukMF’ (*lukM,* GenBank accession: 1262967;* lukF′*, GenBank accession: 1262954), ruminant-specific Staphylococcal complement inhibitor variant (*scn,* Genbank accession*:* ADN53656.1), SaPI encoded ruminant specific vWFbp variant (SaPI *vWFbp*, GenBank accession: HM234507.1), enterotoxin type I (*seI*, GenBank accession: EFC00985.1), enterotoxin L (*seL*, GenBank accession: BAO65763.1), toxic shock syndrome toxin 1 (*tsst-1,* GenBank accession: WP_001035596.1), penicillin-hydrolyzing class A beta-lactamase (*blaZ*, GenBank accession: WP_000733621.1), tetracycline resistance protein type M (*tetM,* GenBank accession: AKI94996.1) and penicillin binding protein 2A (*mecA*, GenBank accession: WP_104447100.1) was determined using LS-BSR output. Genes were identified using a threshold value of BSR of > 0.9 compared to the reference gene. In addition, a heatmap of LS-BSR score of isolates was created for genes annotated as SLL or SA by *prokka*^26^ using the *pheatmap* package^42^ of the R statistical software version v3.5.4^43^*.*

### Statistical analysis

The GLMM analysis was performed using the *lme4* package^44^ of the R statistical software version v3.5.4^43^. The model used clinical manifestation of mastitis (SCM, CM) as outcome variable and CC of *S. aureus* was a fixed effect. The country of origin of isolates was used as a random effect in the model. To reduce the number of levels within the variable CC, CCs represented by < 10 isolates were grouped together into a category ‘Other’. Furthermore, the association between CC and country of origin of mastitis isolates was investigated by Fisher’s exact test and association between genetic cluster and clinical manifestation was investigated using the Pearson's Chi-squared test. Both tests were performed using the R statistical software version v3.5.4^43^*.*

### GWAS

Based on the pangenome analysis in *roary*, GWAS was performed using *scoary* v1.6.16^45^. Default settings of s*coary* were used during our analysis with an initial threshold of naive p < 0.01. Genes that matched our inclusion criteria (BHP p < 0.1; best pairwise comparison p value of p < 0.1) were considered significant results of interest.

## Supplementary information

Supplementary Information.
