# Supplementary material for: Genomic analysis of European bovine Staphylococcus aureus from clinical versus subclinical mastitis
Source: Sci Rep. 2020 Oct 23;10:18172. doi: 10.1038/s41598-020-75179-2 (PMC7584570; doi:10.1038/s41598-020-75179-2)
Supplement: Supplementary file 1 — Supplementary Information. [file 41598_2020_75179_MOESM1_ESM.docx]

**Genomic analysis of European bovine *Staphylococcus aureus* from clinical versus subclinical mastitis**

Jurriaan Hoekstra^1,*^, Aldert L. Zomer^2^, Victor P.M.G. Rutten^2^, Lindert Benedictus^1^, Arjan Stegeman^1^, Mirlin P. Spaninks^1^, Torben W. Bennedsgaard^3,+^, Andrew Biggs^4,+^, Sarne De Vliegher^5,+^, Demetrio Herrera Mateo^6,+^, Reglindis Huber-Schlenstedt^7,+^, Jørgen Katholm^8,+^, Péter Kovács^9,+^, Volker Krömker^10,+^, Guillaume Lequeux^11,+^, Paolo Moroni^12,+^, Luís Pinho^13,+^, Sebastian Smulski^14,+^, Karlien Supré^15,+^, Jantijn M. Swinkels^16,+^, Mark A. Holmes^17^, Theo J. G. M. Lam^1, 18,+^ and Gerrit Koop^1,+^

1. Department Population Health Sciences, Faculty of Veterinary Medicine, University of Utrecht, The Netherlands
2. Department of Basic Sciences, Faculty of Veterinary Medicine, Utrecht University, The Netherlands
3. Department of Animal Science, Aarhus University, Denmark
4. The Vale Veterinary Group, Tiverton, United Kingdom
5. Department of Obstetrics, reproduction and herd health, Faculty of Veterinary Medicine, University of Ghent
6. **Q-Llet SLP, Seva (Barcelona), Spain**
7. Bavarian Animal Health Service, Poing, Germany.
8. DNA Diagnostic, Risskov, Denmark
9. University of Veterinary Medicine Budapest, Hungary
10. Department of Veterinary and Animal Sciences, University of Copenhagen, Denmark.
11. LABOCEA, Fougères, France
12. Department of Veterinary Medicine, University of Milan, Italy
13. Department of Veterinary Clinics, Abel Salazar Biomedical sciences Institute, University of Porto, Portugal
14. Department of Internal Diseases and Diagnosis, Faculty of Veterinary Medicine and Animal Science, Poznan University of Life Science, Poland
15. MCC-Vlaanderen, Lier, Belgium
16. MSD Animal Health, Boxmeer, The Netherlands
17. Department of Veterinary Medicine, University of Cambridge, UK.
18. Royal GD, Deventer, The Netherlands

+ These authors are members of the European Mastitis Panel

* Correspondence to [j.hoekstra1@uu.nl](mailto:j.hoekstra1@uu.nl)

**Supplementary Materials**


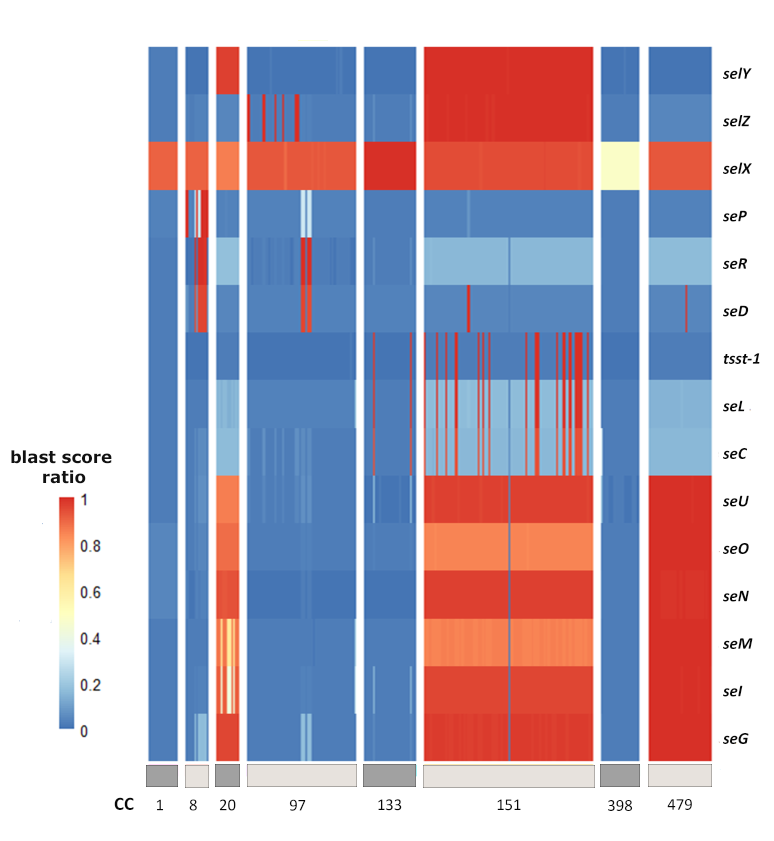


**Supplementary Figure 1.**

A heatmap of BLAST score ratio^25^ (BSR) of genes annotated as Staphylococcal enterotoxins (SEs) by *prokka^26^* of bovine *S. aureus* isolates obtained from clinical and subclinical mastitis cases in 11 European countries calculated using the large-scale BLAST score ratio (LS-BSR) pipeline^25^. Heatmap was visualized using the *pheatmap* package^42^ of R statistical software version 3.5.4^43^. Dark and light grey shading displays Clonal Complex (CC) and only *S. aureus* isolates belonging to CCs with n > 10 in our collection are presented in this heatmap.


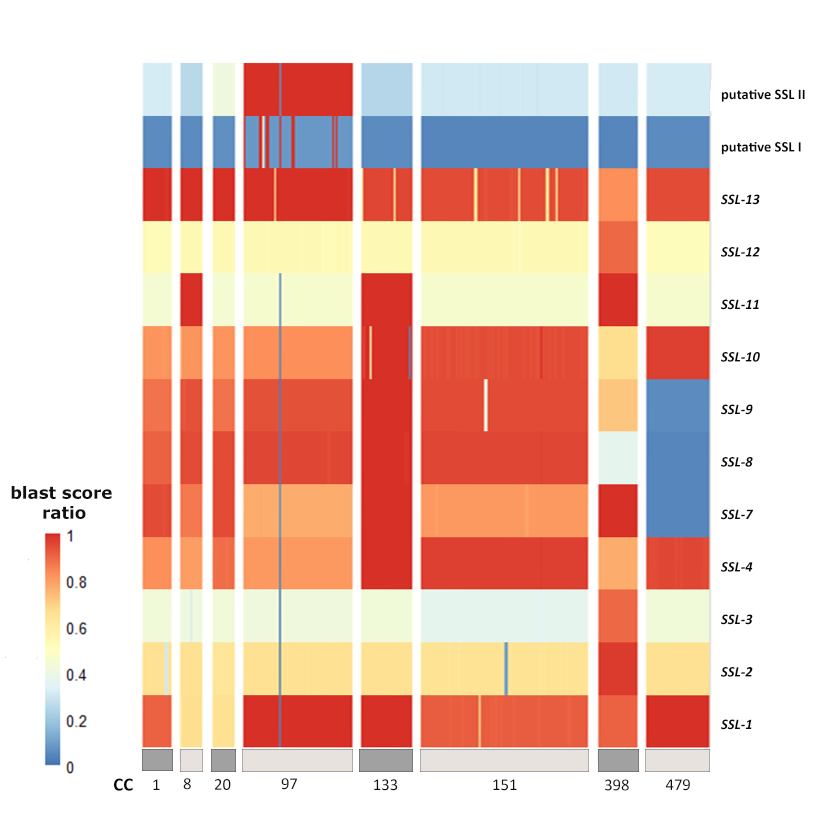


**Supplementary Figure 2.**

A heatmap of BLAST score ratio^25^ (BSR) of genes annotated as Staphylococcal Superantigen like proteins (SLLs) by *prokka^26^* of bovine *S. aureus* isolates obtained from clinical and subclinical mastitis cases in 11 European countries calculated using the large-scale BLAST score ratio (LS-BSR) pipeline^25^. Two yet unnamed SLLs are labeled as putative SLL I (GenBank reference: WP_143564871.1) and putative SLL II (GenBank reference WP_124375191). Heatmap was visualized using the *pheatmap* package^42^ of R statistical software version 3.5.4^43^. Dark and light grey shading displays Clonal Complex (CC) and only *S. aureus* isolates belonging to CCs with n > 10 in our collection are presented in this heatmap.


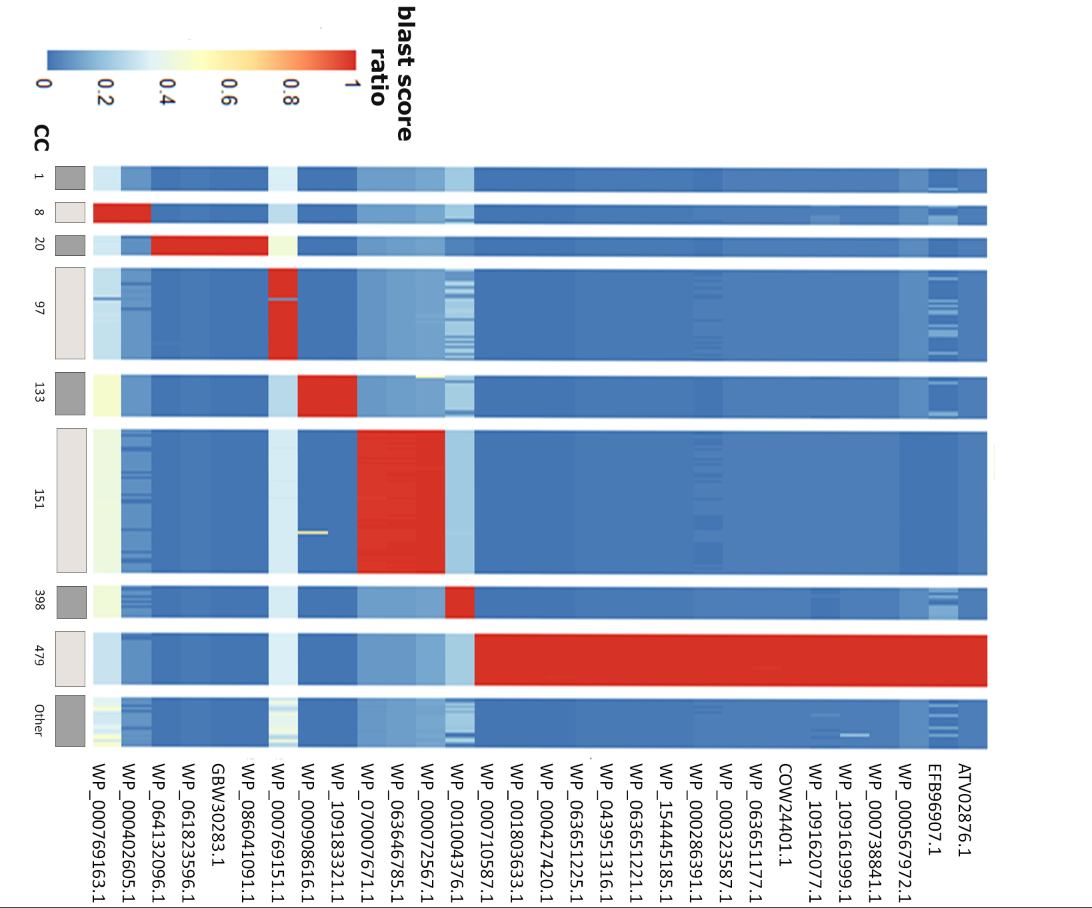


**Supplementary Figure 3.**

A heatmap of BLAST score ratio^25^ (BSR) and GenBank Reference of Clonal complex (CC) exclusive genes of bovine *S. aureus* isolates obtained from clinical and subclinical mastitis cases in 11 European countries calculated using the large-scale BLAST score ratio (LS-BSR) pipeline^25^. Heatmap was visualized using the *pheatmap* package^4^ of R statistical software version 3.5.4^43^. Dark and light grey shading displays Clonal Complex (CC) and isolates belonging to CCs with n > 10 (CC9, CC50, CC5, CC49, CC7, CC45, CC101, CC20, CC30 and CC425.

| **Associated CC** | **Predicted function of gene-encoded Protein** | **GenBank Reference** |
| --- | --- | --- |
| CC8 | SSL-11 variant | WP_000769163.1 |
| CC8 | hypothetical protein | WP_000402605.1 |
| CC20 | site-specific DNA-methyltransferase | WP_064132096.1 |
| CC20 | hypothetical protein | WP_061823596.1 |
| CC20 | hypothetical protein | GBW30283.1 |
| CC20 | DEAD/DEAH box helicase | WP_086041091.1 |
| CC97 | hypothetical protein | WP_000769151.1 |
| CC133 | AAA family ATPase | WP_000908616.1 |
| CC133 | ATP-dependent helicase | WP_109183321.1 |
| CC151 | restriction endonuclease subunit S | WP_070007671.1 |
| CC151 | hypothetical protein | WP_063646785.1 |
| CC151 | restriction endonuclease subunit S II | WP_000072567.1 |
| CC398 | DUF5085 family protein | WP_001004376.1 |
| CC479 | Gfo/Idh/MocA family oxidoreductase | WP_000710587.1 |
| CC479 | polyketide synthase | WP_001803633.1 |
| CC479 | AMP-binding protein | WP_000427420.1 |
| CC479 | salicylate synthase | WP_063651225.1 |
| CC479 | DNA (cytosine-5-)-methyltransferase | WP_043951316.1 |
| CC479 | ABC transporter ATP-binding protein | WP_063651221.1 |
| CC479 | hypothetical protein | WP_154445185.1 |
| CC479 | hypothetical protein | WP_000286391.1 |
| CC479 | 4'-phosphopantetheinyl transferase superfamily protein | WP_000323587.1 |
| CC479 | Eco47II family restriction endonuclease | WP_063651177.1 |
| CC479 | transmembrane component of ECF transporter | COW24401.1 |
| CC479 | helix-turn-helix domain-containing protein | WP_109162077.1 |
| CC479 | YqcI/YcgG family protein | WP_109161999.1 |
| CC479 | MptD family putative ECF transporter S component | WP_000738841.1 |
| CC479 | hypothetical protein | WP_000567972.1 |
| CC479 | hypothetical protein | EFB96907.1 |
| CC479 | Iron aquisition yersiniabactin synthesis enzyme | ATV02876.1 |
|  |  |  |

**Table S1.**

Predicted function and GenBank reference of Clonal complex (CC) exclusive genes among 276 S. aureus isolates obtained from bovine clinical and subclinical mastitis in 11 European countries.
